# Chemical Differences in Environmental Films Collected on Surfaces with Different Hydrophilicity

**DOI:** 10.1021/acsearthspacechem.4c00170

**Published:** 2024-11-14

**Authors:** Jessica L. DeYoung, Uchechukwu Grace Akporere, Zezhen Cheng, Swarup China, Gregory W. Vandergrift, Christopher R. Anderton, Yadong Zhou, Zihua Zhu, Scott K. Shaw

**Affiliations:** † University of Iowa, Iowa City, Iowa state 52242, United States; ‡ Environmental Molecular Sciences Laboratory, Pacific Northwest National Laboratory, Richland, Washington 99352, United States

**Keywords:** atmospheric deposition, particles, environmental film, hydrophobic surface, grime

## Abstract

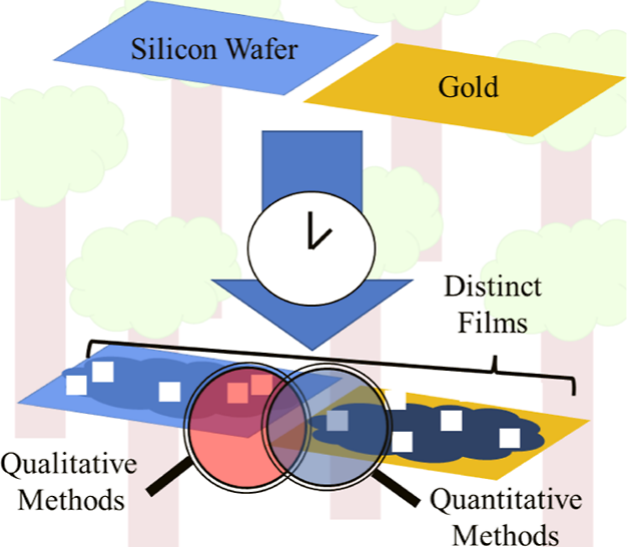

Environmental films form when airborne particles and molecular species adsorb on solid surfaces. Recent studies have characterized these films but overlook how collection methods and host-surface character (orientation, chemical functionality, or height) change the deposition process. In this work, environmental films are collected at a rural location on gold and silicon surfaces (water contact angles of ca. 57° and <1°, respectively) to determine how the different substrate changes the properties of the accumulated environmental film. Results show that gold surfaces have a homogeneous distribution of film mass across the surface, while silicon surfaces collect films with irregular patchy domains. The two surfaces also develop different surface coverages and particle number densities, and the particles’ packing arrangements are quantified by analyzing nearest-neighbor distances. Computer-controlled scanning electron microscopy with energy-dispersive X-ray spectroscopy suggests that, despite morphological differences, larger (>5 μm) particles have similar elemental compositions. Minor variations are observed at smaller particle sizes (∼5 μm), which include carbon-rich particles primarily attributed to pollen or biotic activity. Chemical analysis shows the presence of nitrate and sulfate, as well as heterogeneous cation pools on the surfaces.

## Introduction

Environmental films are composed of particles, organic molecules, dust, and minerals that accumulate on impervious surfaces over time.^1−3^ The films’ chemical composition has been reported to reflect local environmental sources.^4,5^ This includes molecules of interest such as phthalates and polychlorinated biphenyls (PCBs).^6^ Understanding the impact of environmental films on human and environmental health as well as material performance and processes such as corrosion is complex and has been a topic of limited investigation for more than 30 years.^1,7^

Significant research has been conducted on how the chemical composition of films changes with season and location.^5,8−10^ Generally, ca. 6–10% of film mass is defined as organic^10^ which may act as an adhesive for particle matter on the surface, laying a foundation for larger mass accumulations.^10,11^ The remaining fraction is broadly classified as inorganic species (salts, minerals, and an aqueous film phase), which typically accounts for the balance (90–94%) of the films’ mass.^3,9,10,12^ The mass of microbes or biological species, such as spores, is not typically identified or classified but would likely be contained within the “organic” fraction based on common analysis methods. Microorganisms like fungi and bacteria can change the environmental impact of the film, where fungal colonies may work to aggregate particles within a film, for example.^13^ Importantly, the film compositions reported in the literature display wide variations in these broad classes of organic and inorganic, suggesting that film composition is subject to many factors including properties of the substrate. Mass accrual rates for a film depend on the physical form of the species present and specifically the ability for these particles, molecules, or other moieties to interact with the clean host substrate (as well as any material that might already be entrained there).

Bulk chemical analysis of surface films via wash-off methods is valuable, but these approaches overlook the surface physical morphology or roughness changes induced by particles in the film. Recent studies have shown that the film components have a more particle-like nature than originally thought.^14^ The heterogeneity and roughness added by particles is important because it affects a film’s capacity to adsorb and concentrate species of interest, like PCBs or polycyclic aromatic hydrocarbons.^15^

While the chemical processes and physical interactions that lead to film formation have been studied in the past, most of the results have focused on gas phase concentrations of molecular species or deposition velocities of particles.^16^ Our group has recently highlighted the changes in film formation due to host-surface orientation.^17^ Importantly, it is clear that the mass accumulation processes of environmental films are dynamic and variable depending on myriad local environmental factors, as well as the physical and chemical properties of the solid substrate supporting the film (the so-called “host surface”). Particle retention is dependent on the foundation established by the existing host surface and film components. Given that different surface chemistries have different capacities for volatile organic carbon partitioning, particle adsorption, and biotic activity, we hypothesize that environmental films on different host surfaces would be physically and chemically unique.

This study investigates if a surface’s hydrophilicity changes the physical and chemical properties of the formed films. Our primary focus is to characterize the spatial distribution, size, and chemical composition of films developed on substrates with different hydrophilicity. A combination of bright-field microscopy and computer-controlled scanning electron microscopy with energy-dispersive X-ray spectroscopy (CCSEM-EDS) was used. In addition, analysis of the chemical and physical forms of the films was studied using time-of-flight secondary ion mass spectrometry (ToF-SIMS). The results presented here have implications for understanding host-surface characteristics in studies involving environmental films and grime.

## Experimental Section

### Safety

No unexpected or unusually high safety hazards were encountered.

### Surface Preparation and Details

We studied two sample types. The first was a borosilicate glass slide coated with gold (Aldrich) with a roughness of ∼100 nm. The second surface was a polished ∼525 μm-thick silicon wafer with a thermally grown oxide layer of 100 nm and surface roughness <5 nm (University Wafer). All substrates were cut using a diamond edge pen to surface areas of 3–6 cm^2^ and cleaned prior to exposure using fresh piranha solution. Note: Piranha solution is a highly reactive mixture of sulfuric acid and hydrogen peroxide which requires strict safety measures and appropriate PPE. The surfaces were rinsed with ultrapure water, sonicated in ultrapure water, and rinsed again in ultrapure water (18.2 MΩ/cm^2^, <5 ppb toc). The surfaces were dried in a stream of ultrapure N_2_ and stored in a clean, sealed Petri dish until use. Water contact angles of the freshly cleaned surface were ∼57° for Au-coated covered glass and <1° for the silicon wafer. Sample images of water droplets (ca. 20 μL) on these surfaces are shown in Figure S1.

### Environmental Sampling

The environmental films were collected passively by mounting these clean surfaces to an aluminum plate sampler using adhesive carbon tape (Ted Pella),^12^ as previously reported. The samplers were positioned outside, 2 m from the ground, approximately 6 miles north of the University of Iowa campus (ca. 41°44′05.0″N 91°33′50.0″W) for 2 months (from April 2021 to May 2021). The sampling location was a wooded area protected from pedestrian or vehicle traffic. Host-surface samples were stored in clean, sealed Petri dishes before and after placement. Field blanks were prepared the same way and carried to and from the sampling and analysis locations, but they were not exposed at the sampling site. The field and blank sample surfaces were kept in sealed Petri dishes inside a laboratory desiccator between collection and analysis.

### Bright-Field Microscopy

Bright-field images were taken using a Laxco T40 Series Dual Track LED Stereo Zoom Microscope with a magnification of 95× and white light exposure, with an exposure time of 159 ms adequate to distinguish the collected film particles from the background.

### ImageJ Analysis

Image analysis was conducted to identify particle matter and analyze the morphology. We note that the “particles” analyzed here are limited to roughly ≥10 μm in diameter. The code for this analysis (ImageJ) with an explanation of each step is provided in the Supporting Information. We first analyzed the nearest-neighbor distance. This is a measurement of the minimum distance from one individual particle to another. We then measured the area equivalent diameter (AED). This parameter accounts for differences in the particle morphology and provides a holistic comparison of the sizes without differences of shape convoluting the results. This is shown in eq 1
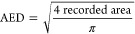
1where AED is reported in μm and the recorded area in μm^2^. Comparing AED with the circularity parameter allowed for a useful analysis of particle morphology. Equation 2 shows this calculation
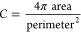
2where C is the circularity (unitless), area is in μm^2^, and perimeter is in μm^2^. Circularity values range from 0 to 1, with values closer to 1 being more circular and values closer to 0 being more branched or elongated. Circularity parameters are shown in detail for sample shapes in Figure S2.

### Total Dissolved Species

After exposure and collection, host surfaces were submerged in 5 mL of water (18.2 MΩ/cm^2^, <5 ppb toc) in conical tubes and sonicated. This method of extraction should isolate ionic and water-soluble species. Total dissolved species (TDS) measurements were made on the resulting solutions with a Mettler Toledo S230 SevenCompact Conductivity Meter. Before the measurements, the meter was calibrated using a 25 μs/cm standard from Millipore Sigma.

### Scanning Electron Microscopy

Scanning electron microscopy (SEM) was conducted by using a FEI Quanta environmental scanning electron microscope with computer-controlled imaging capabilities and an energy-dispersive X-ray detector, which is housed in the Environmental Molecular Sciences Laboratory (EMSL) at the Pacific Northwest National Laboratory. A magnification of 500× was used to focus on particles ranging from 2.5 to 150 μm. The observed size and shape of particles described in this work are based on the projected two-dimensional area. An accelerating voltage of 20.0 kV was used with a current of 0.48 nA. After analyzing ∼60 particles on the Si and Au surfaces, elemental analysis was conducted for C, N, O, Na, Mg, Al, Si, P, S, Cl, K, Ca, Mn, Fe, and Zn. Principal component analysis was used to identify which elements varied the most for the collected films. We found this result by using the loadings of the first three principal components (which accounted for ∼90% of the variance). Elemental mapping was performed using an Oxford EDS detector with a collection time of 20 min.

### Time-of-Flight Secondary Ion Mass Spectrometry

ToF-SIMS measurements were conducted using a TOF.SIMS5 instrument (ION-TOF GmbH) located in the EMSL. A 25 keV Bi_3_^+^ primary ion beam was used to probe the sample surfaces over 500 μm × 500 μm areas. The total Bi_3_^+^ ion dose remained under the static limit (1 × 10^12^ ions/cm^2^) for all measurements. To ensure good secondary ion signal intensity, a low-energy electron flood gun was used to compensate for potential charging. The data was processed using SurfaceLab 6 software. Statistical analysis for the data collected here is described in Figure S3.

### Results and Discussion

#### Bright-Field Imaging Analysis

**Figure 1:**
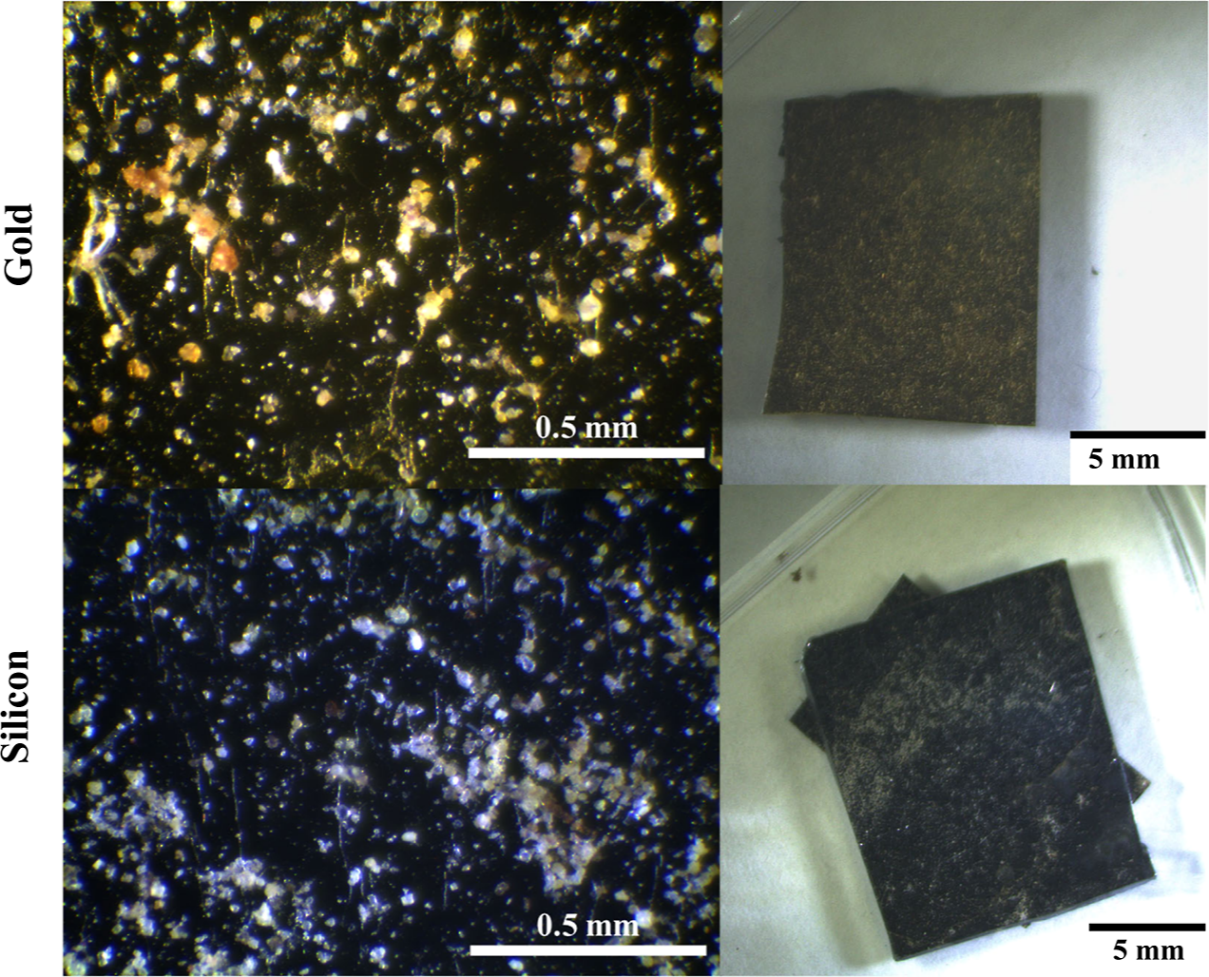
Bright-field microscopy images of the hydrophobic (top) and hydrophilic (bottom) surfaces after the sampling period.

Figure 1 shows representative images of the gold (top) and silicon (bottom) surfaces. There is evidence of leaf stellate (larger branched species), fungal growth (long thin structures across the surfaces), and particles distributed across both host surfaces. Based on the lower-magnification images in Figure 1, it appears that the spatial distribution of particles is different on Au vs silicon surfaces. Specifically, the Au surface has collected a number of particles that are more evenly distributed across the surface than on the silicon surface. Notably, the particle distribution on the silicon was highly heterogeneous, where significantly more particles clustered near the top third of the silicon wafer. This is qualitative evidence that silicon wafers have more heterogeneous domains in comparison to the gold surface. These are the first results of the work suggesting that the morphology of films collected on silicon- and gold-supported films is different.

**Figure 2:**
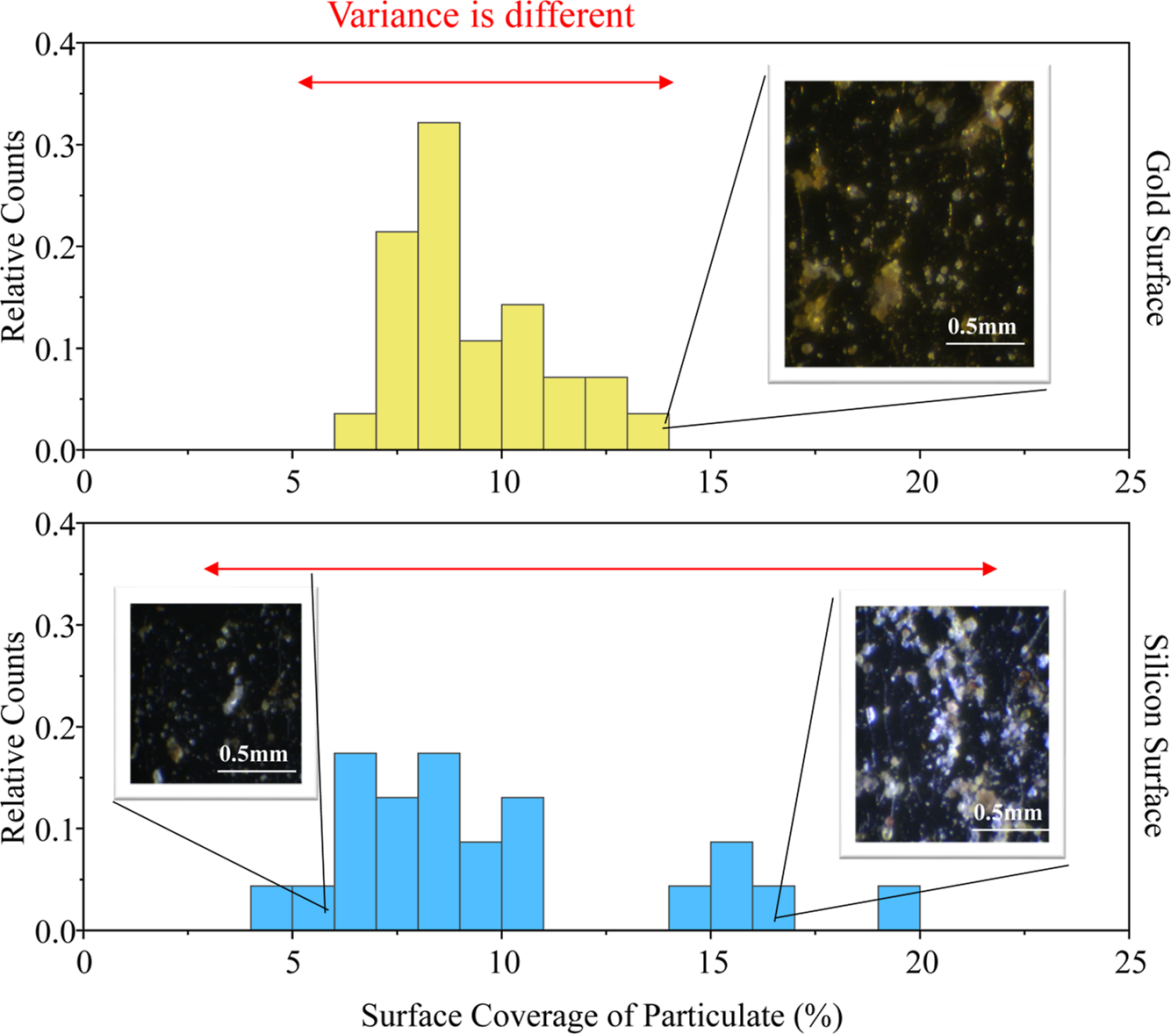
Histograms showing the distribution of surface coverage for *n* = 28 photos for gold and *n* = 23 photos for silicon. Results of the gold surface analysis are shown at the top, while results for the silicon surface analysis are shown at the bottom. There are red arrows to highlight the differences of variance between the two data sets. Images show representative optical microscopy of the domains observed.

We further analyzed the surface coverage of the particles and the number of particles per unit area for each surface (Figures 2 and S4, respectively). The histograms shown for the Au (top, yellow) and silicon (bottom, blue) images are binned results for each image of the surfaces (Au *n* = 28 images, while silicon *n* = 23 images). Images typically represent 1.5 cm^2^ of surface area. The surface coverage analysis in Figure 2 shows that the films formed on Au surfaces have a narrower distribution of calculated surface coverage in comparison to the silicon surfaces. The silicon surfaces show more distinct domains of low or high coverages, while coverage on the Au surfaces is more uniform; this is because silicon surfaces tend to form more aggregates, while Au surfaces allow for a more uniform distribution. These histograms quantitatively illustrate the heterogeneity represented in images shown in Figure 1. Specifically, an important nuance would be overlooked if only averages of the surface coverage metric are reported.

These findings are significant because they show that Au and silicon surfaces collect particles in different ways, seen via the differences in the surface coverage distribution. Our previous results suggest that surface coverage (as measured with bright-field microscopy) scales with roughness (as measured with atomic force microscopy). However, Figure 2 shows that the two surface types present different capacities for film formation and retaining mass, which will significantly affect the physical properties of the film and its effects on substrate performance.^15^ To interpret the details of this observation, we first relate the surface coverage to the particle size distributions. We suspect that fewer large particles or more smaller particles might be contributing to the disparity. To do this, we studied the number of particles per unit area (i.e., density). Figure S4, which provides a histogram of the number of particles per unit area for each surface, shows that the variance of particle number density is insignificantly different between substrate types, but the mean averages are significantly different.

#### Discrete Particle Properties

**Figure 3:**
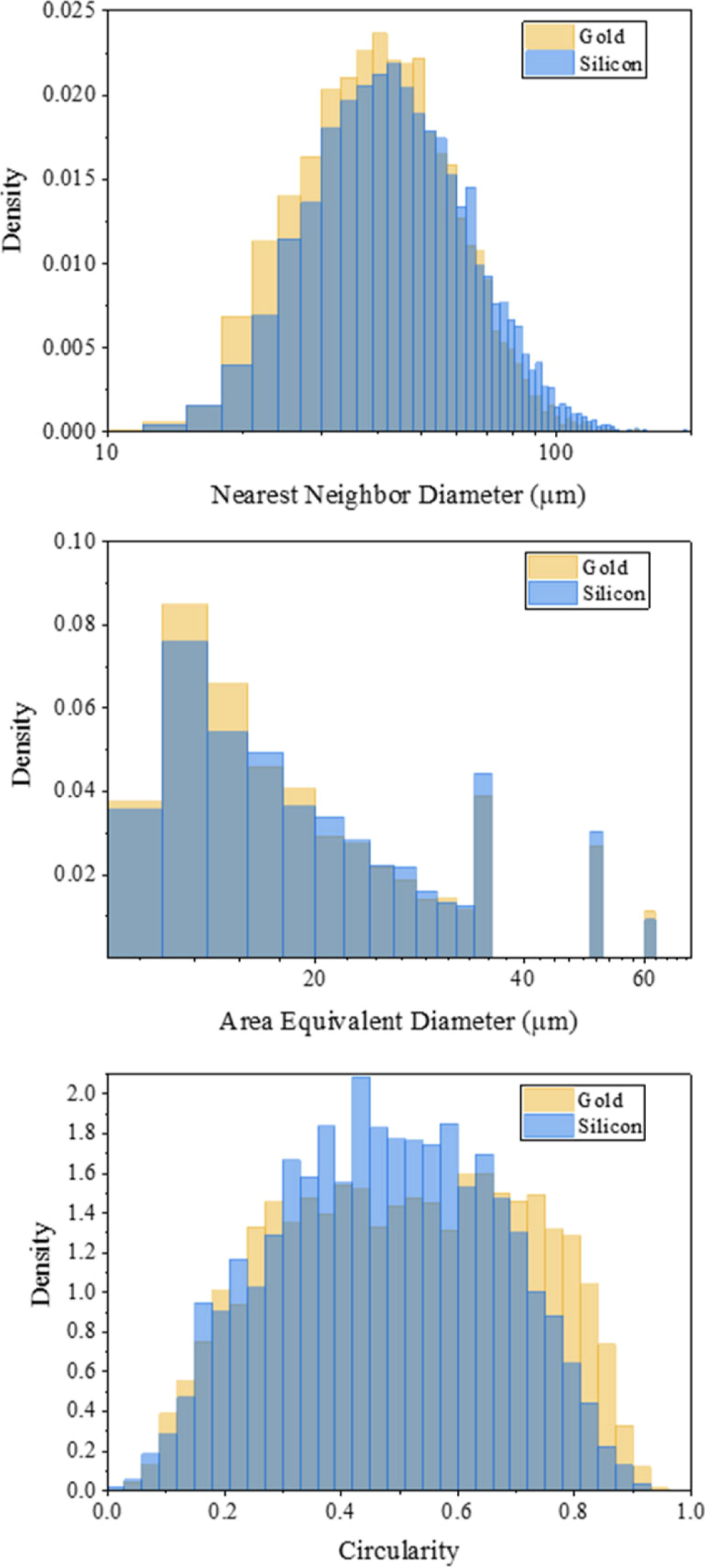
For each particle: nearest-neighbor distance (top), AED (middle), and circularity (bottom). The distribution for the gold surface is shown in yellow, while the distribution for silicon is shown in blue. The *y* axis is the probability density for each. Particles on gold exhibit closer nearest-neighbor distances.

Three metrics to identify differences in the particle morphology and distribution are shown in Figure 3 histograms, which overlay distributions of nearest-neighbor distance, AED, and particle circularity for films developed on Au (yellow) and silicon (blue) surfaces. The number of particles analyzed for the films on Au is 6479, while 4250 particles were analyzed from films on silicon. An alternative representation of the data in Figure 3 is shown by violin plots in Figure S5. In both representations, the differences in the nearest-neighbor distance are most apparent, with the Au surfaces showing shorter distances (closer particles) overall (Figure 3, top). The nearest-neighbor values both show normal distributions. The variance (*F* = 0.77) and average of the data are different (Welch-corrected *T* value of 11.32). The images in Figures 1 and 2 support this qualitatively, and overall, the results suggest that the nearest-neighbor distance can be represented as an average with a homogeneous distribution across the surface despite the aggregate domains. The Au and Si surface particle populations are determined to be statistically different with a Welch-corrected *T* value of 5.23. In addition, the silicon surface also shows more evidence of aggregates due to their increased AED values. This is apparent in Figure 3 at the bottom, which shows a significant shoulder for particles on Au at circularity values approaching 1. This is further emphasized in Figures S5 and S6, both of which show circularity values as violin plots.

While these results are interesting, the AED does not show major differences between the particles accumulated on the two surfaces, and the biggest difference between surfaces is shown in the circularity parameter (*F* = 1.25). The Au surface shows two main types of particle morphologies. The first are at circularity ≈0.7, which are relatively compact, isolated particles. The other is at circularity ≈0.2, attributed to biotic particles with oblong, elongated structures.^13^ This maybe suggestive of distinct particle morphologies, which are uniformly distributed across the surface. In contrast, the silicon surface shows one major particle type centered at circularity ≈0.5. While these trends are interesting, our interpretation is limited without an understanding of the elemental composition of the particles. Figure S2 shows how the circularity is calculated with examples of various shapes.

#### Computer-Controlled SEM

While the particles’ physical morphology and spatial distributions are useful to understand the film’s bulk properties, the particle-level properties are also informative. Because the particles are comprised of either dust (oxygen-rich), minerals (calcium-rich), or biotic particles/pollen (carbon-rich), we see that SEM–EDS data show that the variation in composition is principally due to carbon, oxygen, and calcium. Note, we did not include the measurement of Si in this analysis because both substrates contain this element and it contributed to a large background signal. For easier interpretation, the elemental composition was reduced to the ternary fractions of oxygen, calcium, and carbon. The resulting ternary composition diagram and principal components are shown at the bottom of Figure S7. The distributions show clear groups, suggesting the three particle types as discussed below.

CCSEM analysis limits were set for particles between 2.5 and 150 μm diameter, which is inclusive of the size distribution of particles observed in our optical microscopy which typically ranged from 3 to 30 μm (e.g., the distribution in Figure S5, middle). The data were analyzed to identify any correlations between particle morphology and elemental composition. Each particle was assigned to a particle type based on fractional composition. Identified particle types include biotic activity/pollen (carbon-rich particles), single component dust (oxygen-rich species), and multicomponent dust (calcium-rich particles). One interesting aspect to note is the morphologic diversity of the biotic/carbon-rich particles.

**Figure 4:**
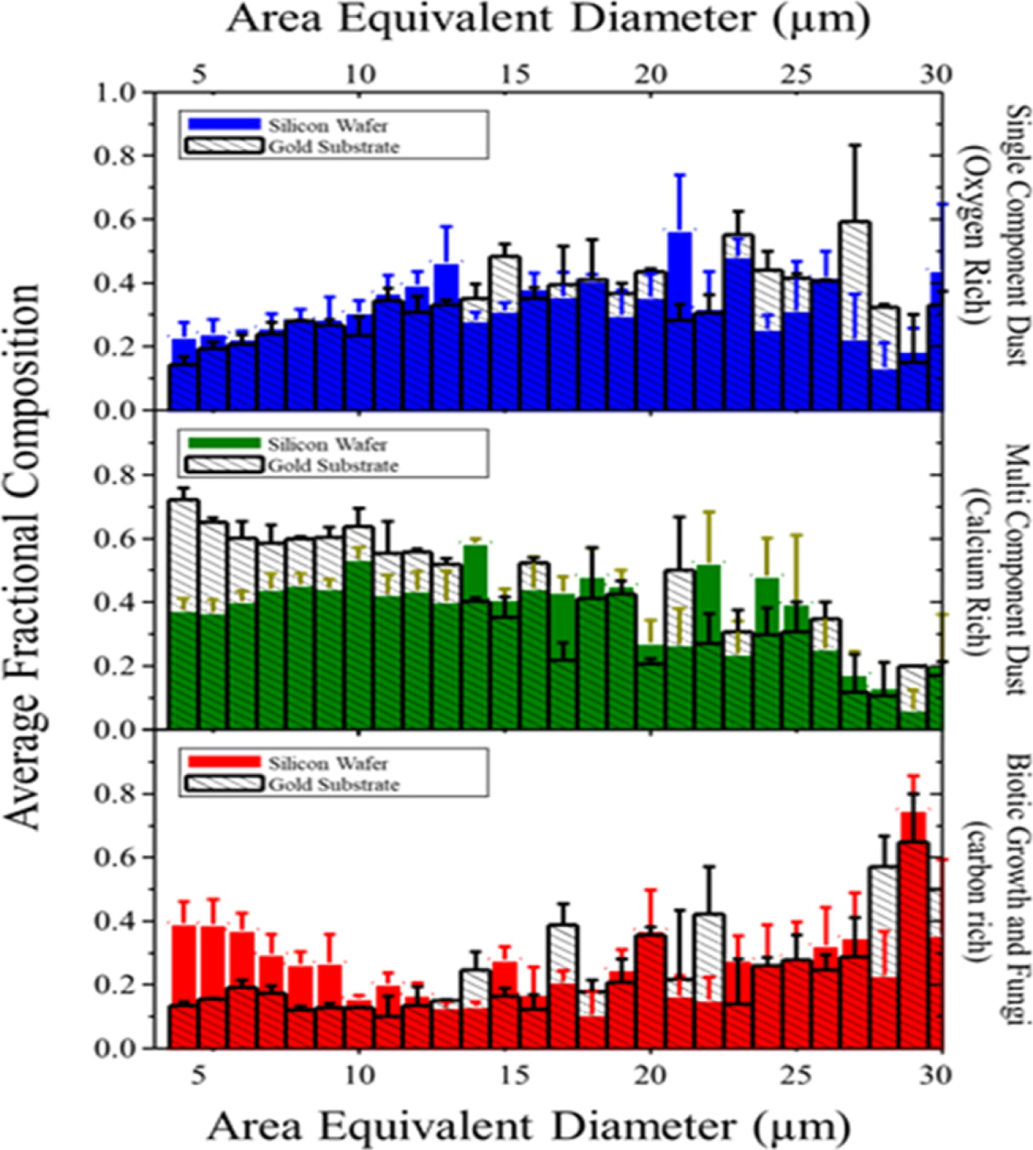
Bar charts showing the fractional composition and the standard deviation for each binned AED. The color bar charts are for the silicon wafer, while the empty bars correspond to the gold surface. Each plot dissects the differences between each of the particle types.

SEM images showing samples of each individual particle type are shown in Figure S8, including intact pollen, ruptured pollen, a fungal moiety, and leaf stellate. The morphology of this leaf stellate has similar shape and size to oak plant stellate found around the Iowa City region. Bar charts, binned by particle size, show fractional composition contribution of each particle type as a function (Figure 4). These results show distributions measured on Au surfaces (gray hashed bars) and silicon surfaces (colored bars). An alternative representation which reports the fractional composition of particles as a function of their size (AED) for the same data set is shown in Figure S9. Our data show a significant difference in these data between the two substrate types. Specifically, the smallest AED particles on silicon contain more biotic particles than on the Au substrate. Both surfaces support an increasing number of oxygen-rich particles as AED values increase to a limit of around 15 μm, at which point the average composition plateaus and decreases slightly for the largest AED particles.

The multicomponent dust particles are the most prevalent on the gold surface at AED values < 15 μm. The drop in numbers of these particles above 15 μm is commensurate with increases in both biotic and single component dust. While Figure S9 represents a single surface of each type, data shown in Figure 4 represents data from two surfaces of each type and two areas of each of these substrates (total of 4 areas sampled). The main differences in the data presented here are that the silicon surfaces seem to have higher mixed-component particles further supporting higher aggregating behavior than the gold counterpart. This finding is expected as dust would be generated from local agriculture activity, road dust, or suspension from other ground-based sources which could change the reactivity or water affinity of the surface.^18^ There is also evidence of pollen and leaf stellate in several different forms which could explain the prevalence of fungal activity at this site, as pollen and stellate could act as a transport for spores or source of nutrients for fungal growth.^3^ The form of the stellate and nearby particles is shown by using SEM-EDS images in Figure S10.

An additional observation at this site is the prevalence of calcium carbonate or calcium oxalate particles. We believe this is due to limestone associated with the regional terrain as a previous study identified similar particle chemistry in samples acquired ca. 5 km from the sampling locations used here.^5^ The analysis suggested a CaCO_3_ species that could contribute to local acid–base chemistry on the surface.^19^ Like the previous observations, these particles are often found in large aggregates (such as those seen in Figure S11). Interestingly, a closer look at the underlying surface suggests that fungal growth could be correlated with particle aggregation, as shown in Figure S12.

#### TDS and Buffering Capacity

**Figure 5:**
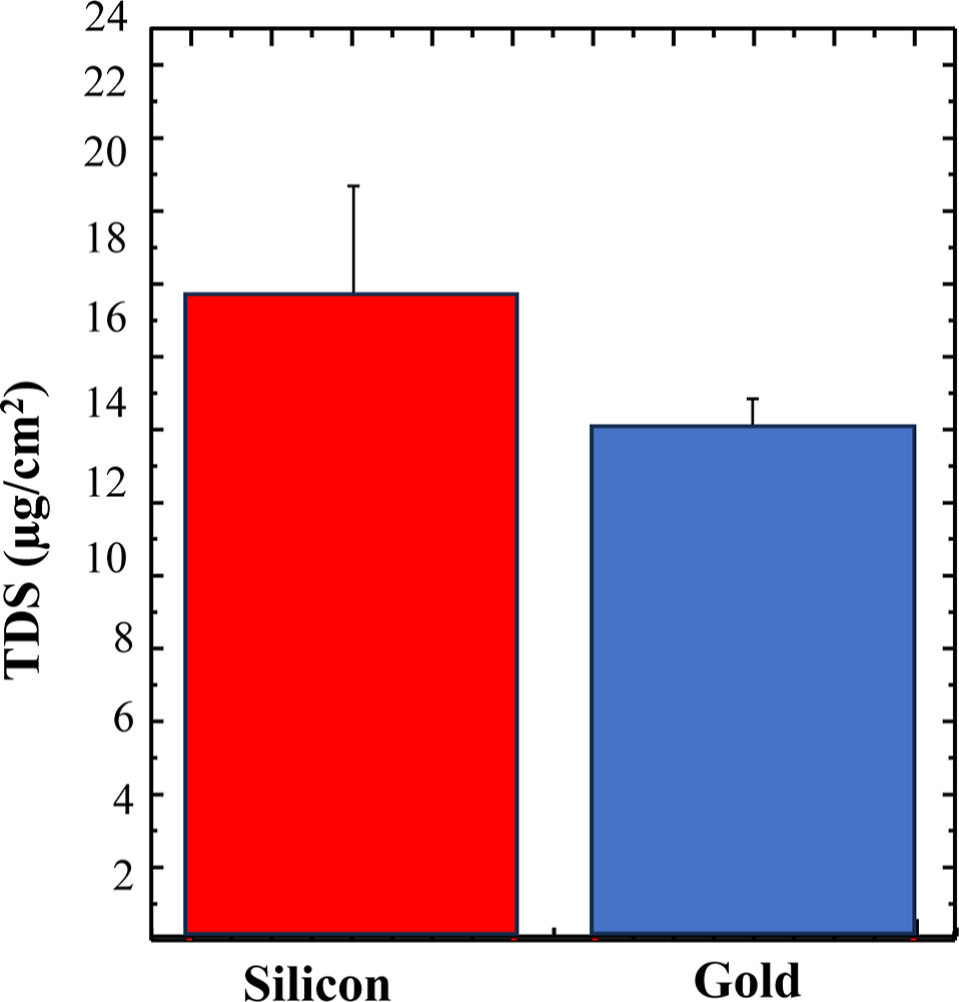
TDSs for the silicon- and gold-supported substrates. The values here are reported with standard error. With these measurements, we have the pH contributions of the two films seeing that the gold substrate has more pH contribution than the silicon substrate. These results support chemical differences of the films supported by each substrate.

To better understand the dissolvable-particle and water-soluble contribution to wastewater, a combination of total dissolved solid (TDS) measurements was used. The results for the TDS analysis are shown in Figure 5 which shows the extracted material mass normalized to the surface area on which the film was collected. Because of limited sample availability, we were only able to measure two replicates of each surface type. The measured TDS values, reported as averages with standard error, are 18 ± 3 μg/cm^2^ for the Au surface and 14.0 ± 0.8 μg/cm^2^ for the silicon surface. Hence, the Au substrate has the capacity to capture and release more accumulated film in comparison to the silicon substrate.

#### Chemical and Spatial Analysis

**Figure 6:**
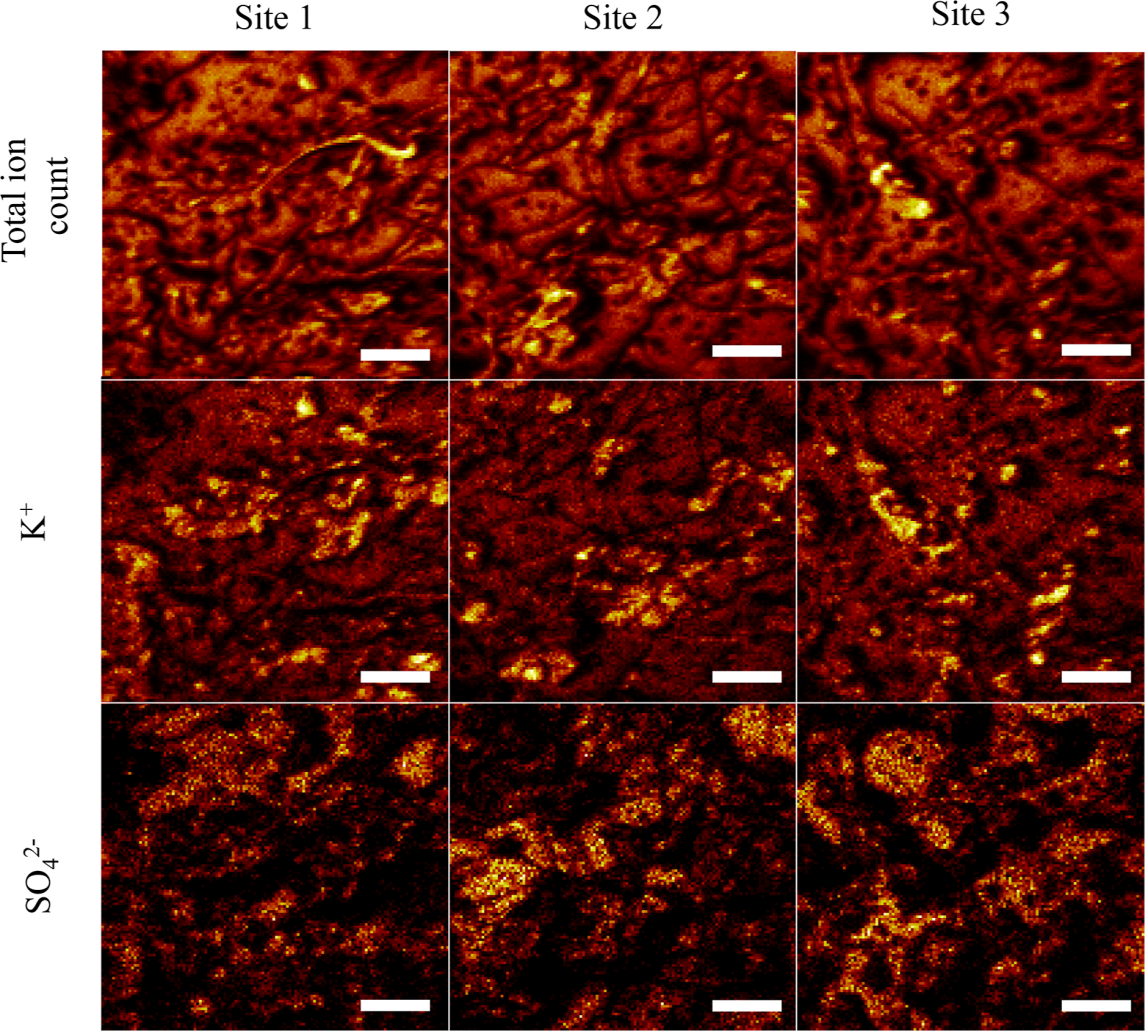
ToF-SIMS images showing three different sites on the gold surface. The three columns represent three sites, while the rows show the distribution of the total ions (top), potassium (middle), and sulfate (bottom). The scale bar is 100 μm.

We noted earlier that differences in the spatial distribution of particles may correlate to inequal chemical profiles across the surface. To test this hypothesis, we used spatially resolved chemical analyses to study the homogeneity of adsorption across the planar surface. Specifically, we show ToF-SIMS imaging data in Figures 6 and S13 to visualize the distribution of sulfate, potassium, sodium, a selected organic fragment (C_26_H_35_O_6_), and nitrate. Noting the distribution, and local concentrations, of these species will help us understand how the films are formed and how they could work to selectively concentrate (micro)nutrient or other chemical species resulting in the heterogeneous character of environmental films. To this end, three replicate sites on the Au surface are shown in Figure 6. This plot shows the total positive ion contributions, potassium distribution, and sulfate distribution. We see that all species, particularly sulfate (Figure 6) and nitrate (Figure S13), show uneven coverages across these surface sites. Ultimately, these results show that environmental films cover the host surfaces within 2 months of placement, suggesting that shorter collection times may be needed to study possible cooperative adsorption mechanisms that lead to the heterogeneity observed.

Additional analysis of the surface with nanospray electrospray ionization mass spectrometry (nano-DESI-MS) was used to characterize some additional molecular species. The nano-DESI-MS data was processed using DECONTOOL^20^ and assigned to a specific molecular formula. The van Krevelen plot is used to display the molecular features in Figure S14. The van Krevelen plot identified 24 individual molecular species, most of which fall under the classification of lipids.^21^

## Conclusions

This manuscript describes implications of the net impact of host surfaces with different hydrophilicity on their subsequent film formation. Our central research question was focused on how particles on surfaces with different hydrophilicities would differ. We found the following:

- Silicon substrates build environmental films with higher surface area.
- Silicon substrates support more particle aggregates which means fewer but larger particles when compared to the Au surfaces.
- There are minor differences in the chemical composition of the particles between surfaces suggesting that the morphology and spatial heterogeneity are the major differences between the host substrates.
- Ionic species that are often attributed to gas phase partitioning are shown to be heterogeneously distributed across the surface implying a significant role of surface films even for “volatile” species.

Our results contribute fundamental insights into host-surface interactions and their effects on accumulated environmental films. Applications of these results contribute to modeling particle and gas phase partitioning to surfaces and suggest a significant role for the host surface in determining the composition and morphology of the resulting environmental film.

## Supporting Information Available

Supplementary experimental details, methods for statistical analyses, and additional microscopic images of the films and plots to support the findings reported here (PDF)
