# Supplementary material for: Chemical Differences in Environmental Films Collected on Surfaces with Different Hydrophilicity
Source: ACS Earth Space Chem. 2024 Nov 14;8(12):2411–9. doi: 10.1021/acsearthspacechem.4c00170 (PMC11664647; doi:10.1021/acsearthspacechem.4c00170)
Supplement: Supplementary file 1 — sp4c00170_si_001.pdf [file sp4c00170_si_001.pdf]

SUPPPORTING INFORMATION

Title: Chemical Differences in Environmental Films collected on Surfaces with Different Hydrophilicity

Authors:

- Jessica L DeYoung, University of Iowa, Iowa city, Iowa state, 52242, USA, ORCID: 0000-0001-7484-4968
- Uchechukwu Grace Akporere, University of Iowa, Iowa city, Iowa state, 52242, USA, ORCID: 0000-0003-0192-8911
- Zezhen Cheng, Environmental Molecular Sciences Laboratory, Pacific Northwest National Laboratory, Richland, Washington 99352, USA, ORCID: 0000-0001-6320-4519
- Swarup China, Environmental Molecular Sciences Laboratory, Pacific Northwest National Laboratory, Richland, Washington 99352, USA, ORCID: 0000-0001-7670-335X
- Gregory W. Vandergrift, Environmental Molecular Sciences Laboratory, Pacific Northwest National Laboratory, Richland, Washington 99352, USA, ORCID: 0000-0002-8962-9897
- Christopher R. Anderton, Pacific Northwest National Laboratory, Environmental Molecular Science Laboratory, Richland, Washington, 99352, USA, ORCID: 0000-0002-6170-1033
- Yadong Zhou, Pacific Northwest National Laboratory, ORCID: 0000-0002-9012-7536
- Zihua Zhu, Environmental Molecular Sciences Laboratory, Pacific Northwest National Laboratory, Richland, Washington 99352, USA, ORCID: 0000-0001-5770-8462
- Scott K. Shaw*, University of Iowa, Iowa city, Iowa state, 52242, USA, ORCID: 0000-0003-3767-3236

*denotes corresponding author

Corresponding author email address: Scott-k-shaw@uiowa.edu

Keywords: atmospheric deposition, particles, environmental film, hydrophobic surface, grime

Table of Contents:

**Figure S1:**	Images showing the contact angle of the two substrates	S3
**Figure S2:**	Images showing calculation and numbers to explain how circularity is calculated	S4
**Figure S3:**	Details on statistical analysis	S5
**Figure S4:**	Histograms showing number of particles observed	S6
**Figure S5:**	Violin plots showing three parameters analyzed for surfaces in this study.	S7
**Figure S6:**	Violin plots showing the distribution of area equivalent diameter and circularity for each surface replicate	S8
**Figure S7:**	Plots showing the PCA analysis and reduced form of the elemental fractions of the particulate.	S9
**Figure S8:**	SEM images showing particulate categorized as one of the three types	S10
**Figure S9:**	Histogram showing the variability of four replicates of the CCSEM data	S11
**Figure S10:**	SEM and EDS mapping of leaf stellate and aggregates	S12
**Figure S11:**	SEM and EDS mapping of inorganic particles on silicon wafers	S13
**Figure S12:**	SEM and EDS mapping of biotic moieties observed	S14
**Figure S13:**	ToF-SIMS mapping of ions of interest on the gold surface	S15
**Figure S14:**	Van Krevelen plot showing the identified species using Nano-DESI	S16

Figure S1: Images showing the contact angle of the two substrates.

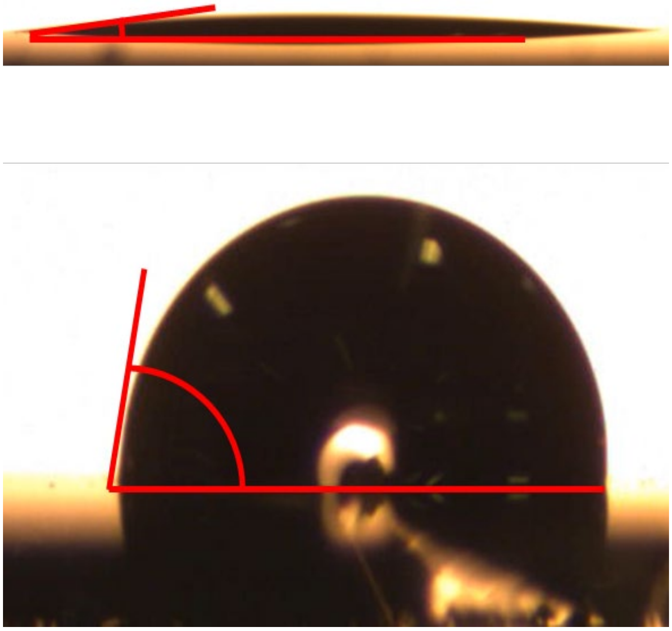

Figure S1: Images of water droplets on the clean host surfaces - silicon wafer (top) with contact angle <1° and gold surface (bottom) with contact angle ~57°. The contact angle measurement is outlined in red.

Figure S2: Images showing calculation and numbers to explain how circularity is calculated.

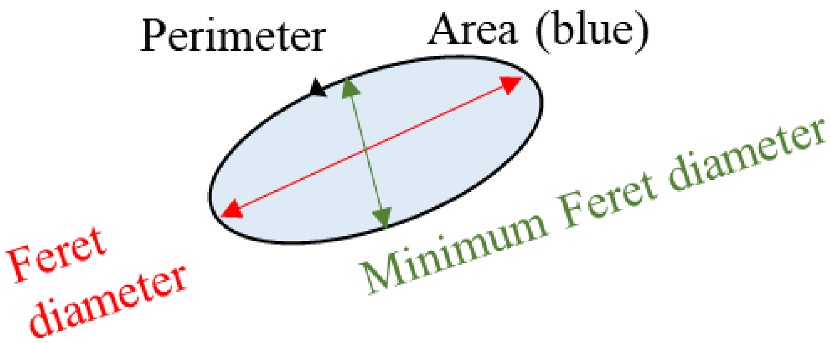

$$\text{Elongation} = (D_{max} - D_{min})/(D_{max} + D_{min})$$

$$\text{Circularity} = 4\pi \text{Area}/\text{Perimeter}^2$$

**Shape**	**Circularity**	**Shapes**
Circle	0.907	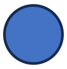
Cloud	0.752	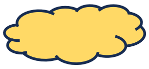
Rectangle	0.573	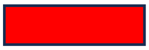
Lightning	0.276	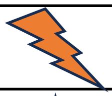
Star	0.182	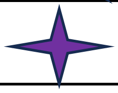

Figure S2: Images showing calculation and numbers to explain how circularity is calculated using perimeter, area and minimum and maximum ferret diameter.

S3: Details on statistical analysis

The number of replicates of the work presented here ranges from two (number of surfaces for TDS analysis) to greater than a thousand (numbers of particles analyzed on a surface). Given the wide range of methods used in this work and the significant heterogeneity observed in the real samples, the authors want to carefully describe limitations on the "certainty" of calculated values as dictated by the number of replicates to avoid any false impressions of over confidence in our data. For example, the silicon surfaces analyzed here show large domains (mm^2) that have different morphologies than adjacent, homogeneous area. To mitigate this, some of the larger, more heterogeneous datasets are represented as histograms with bin sizes determined by the Freedman Diaconis equation.[1] Meanwhile, individual particle data is shown using violin plots. All statistical testing was performed in OriginPro software. Our approach first tested to the variation between datasets, and then tested for significant differences using a two-sample t-test. If the samples show similar variance, the results are interpreted as is, but if the variance is different, the Welch's results are used. For variance comparison an F value is listed. This is the ratio of the two variances, so a value closer to 1 suggests that the variances are more similar. We also list the standard t-statistic values.[2]

1. Diaconis, P., and D. A. Freedman. "Cauchy's Equation and De Finetti's Theorem." Scandinavian Journal of Statistics 17, no. 3 (1990): 235–49. http://www.jstor.org/stable/4616171.
2. Bahadur, R. R. "A Property of the T-Statistic." Sankhyā: The Indian Journal of Statistics (1933-1960) 12, no. 1/2 (1952): 79–88. http://www.jstor.org/stable/25048116.

Figure S4: Histogram showing number of particles observed

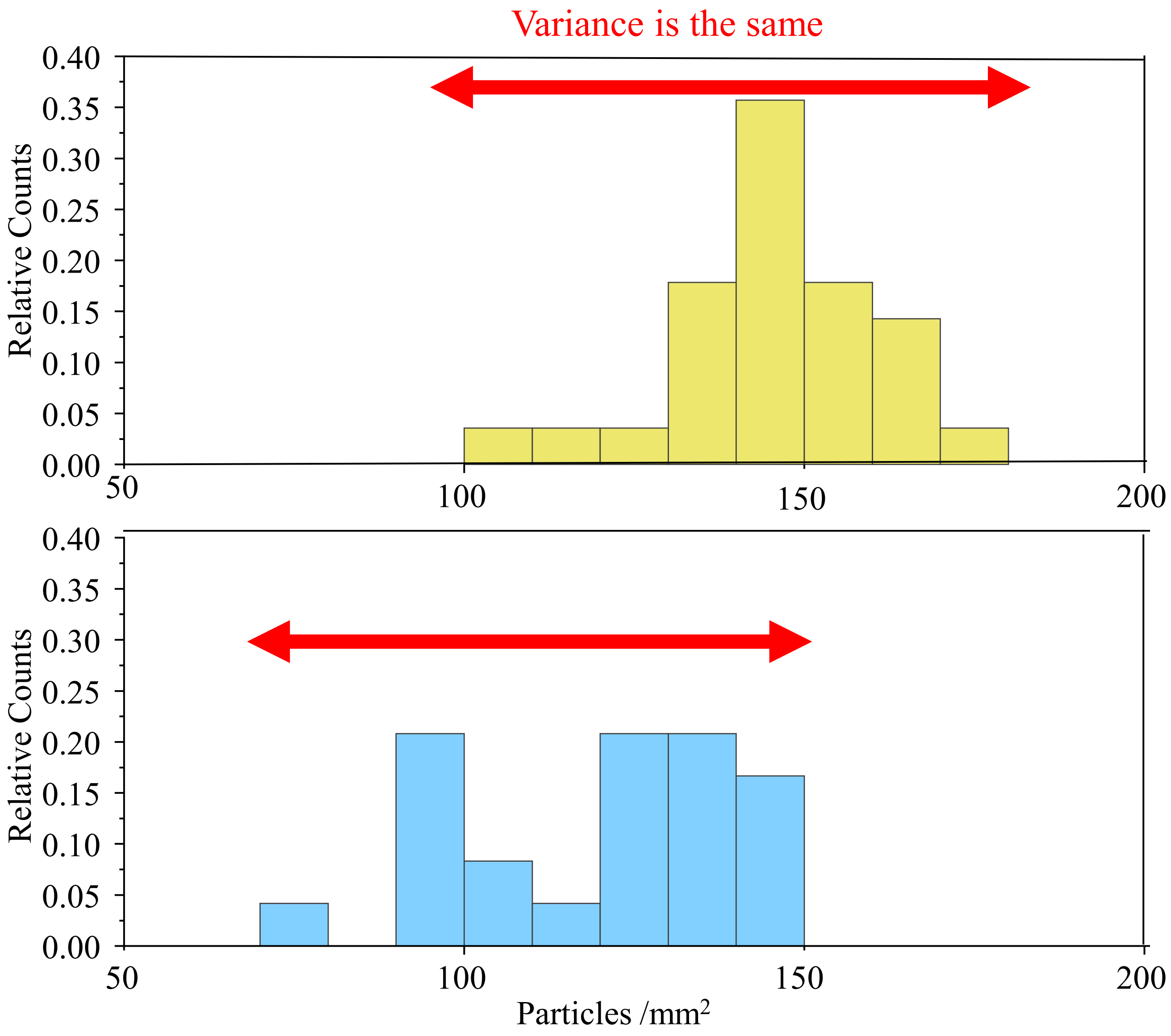

Figure S4: Histograms showing the binned results of the number of particles per unit (number density) area for the gold (top) and silicon (bottom) surfaces. Red arrows are added to compare the variances of the two datasets.

We find that the variance of the two datasets is not significantly different (F=0.67), but the analysis of the data sets shows significant differences between the two surfaces (t statistic= 4.85). The results do not change when separating the different domains mentioned above (F=0.63 and t statistic=5.27). Apart from showing a significantly different surface coverage, the silicon surface shows fewer particles/mm^2. This supports the hypothesis (shown qualitatively in Figure 1) that the film materials aggregate more on the silicon surface in comparison to the gold surface. In contrast to the surface coverage, statistical analysis of the data in Figure 3 shows that the average values for the films on the gold surface and the silicon surface are not the same.

Figure S5: Violin plots showing three parameters analyzed for surfaces in this study.

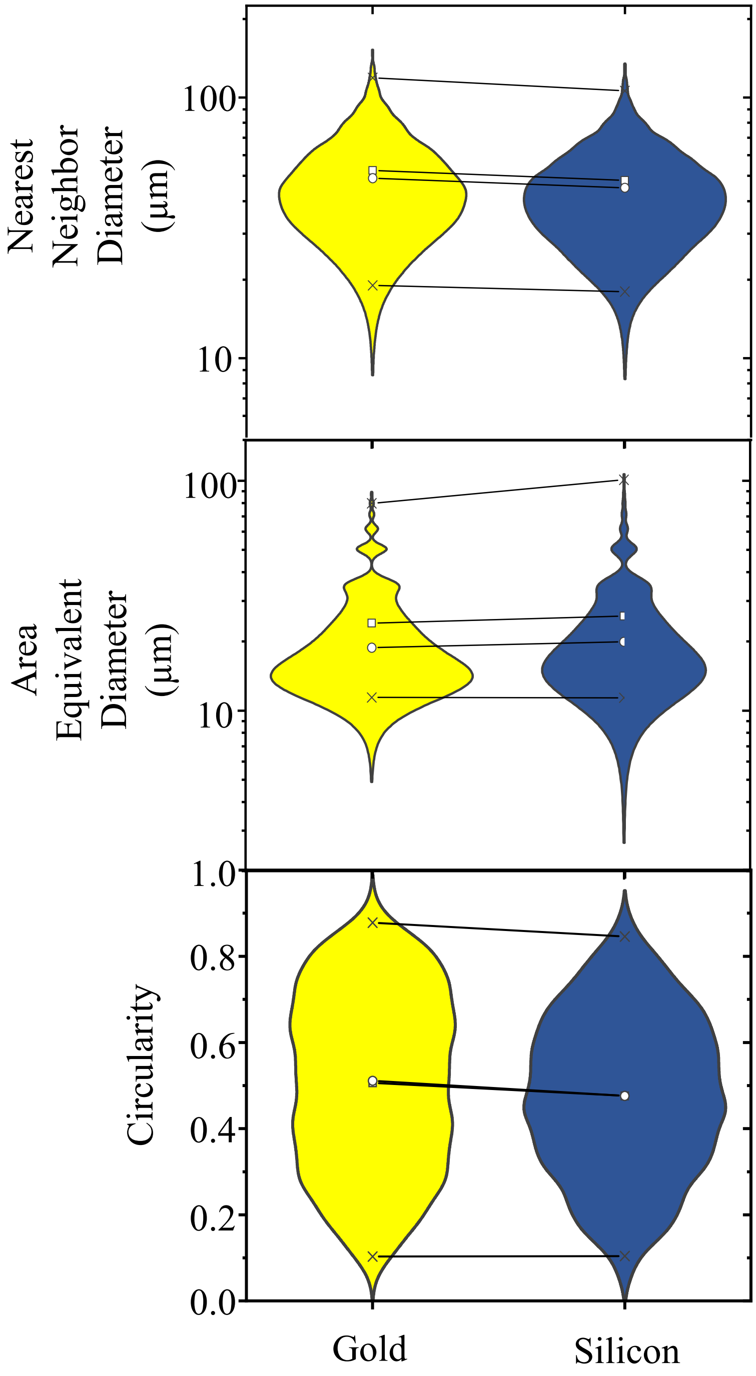

Figure S5: violin plots showing the data in **Figure 3**. The top plot shows the nearest neighbor diameter, the middle the area equivalent diameter, and the bottom the circularity. The lines connect the 99th percentile (top x), mean (circle), medium (square), and first percentile (bottom x). The mean and median lines for the circularity plot overlap.

Figure S6: Violin plots showing the distribution of area equivalent diameter and circularity for each surface replicate

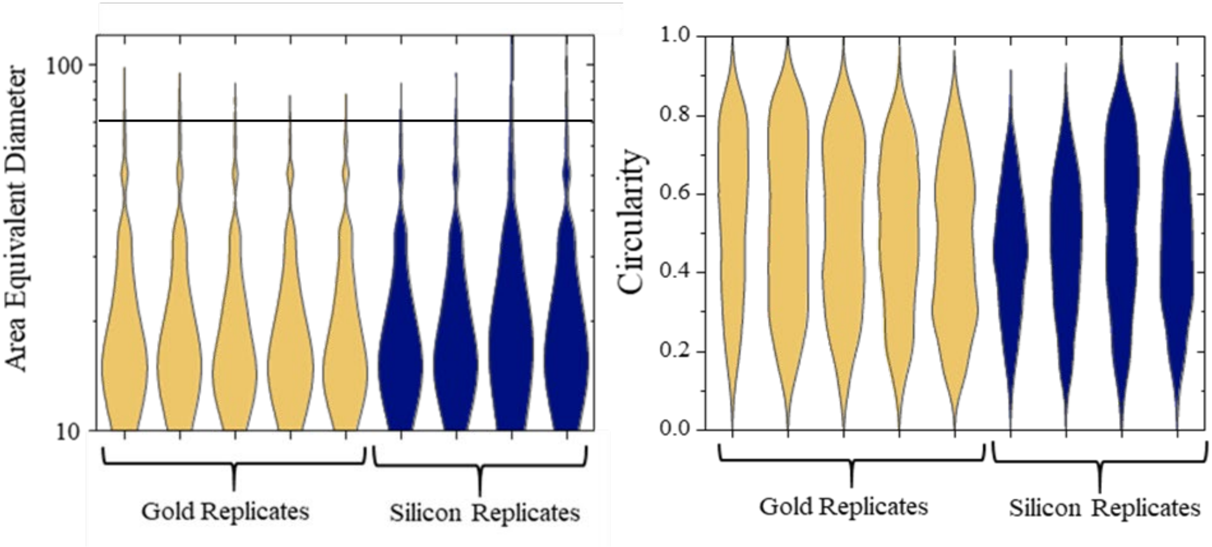

Figure S6: violin plots showing the replicates for each surface analyzed of the gold (yellow violins) and silicon (blue violin). The left plot shows distributions for the area equivalent diameter and the right plot shows the distributions for the circularity. The left plot has a line designating the area equivalent diameter associated with particulate that do not reach "true suspension" (AED > 70 µm) and can be designated as aggregates on the surface.

Figure S7: Plots showing the PCA analysis and reduced form of the elemental fractions of the particulate.

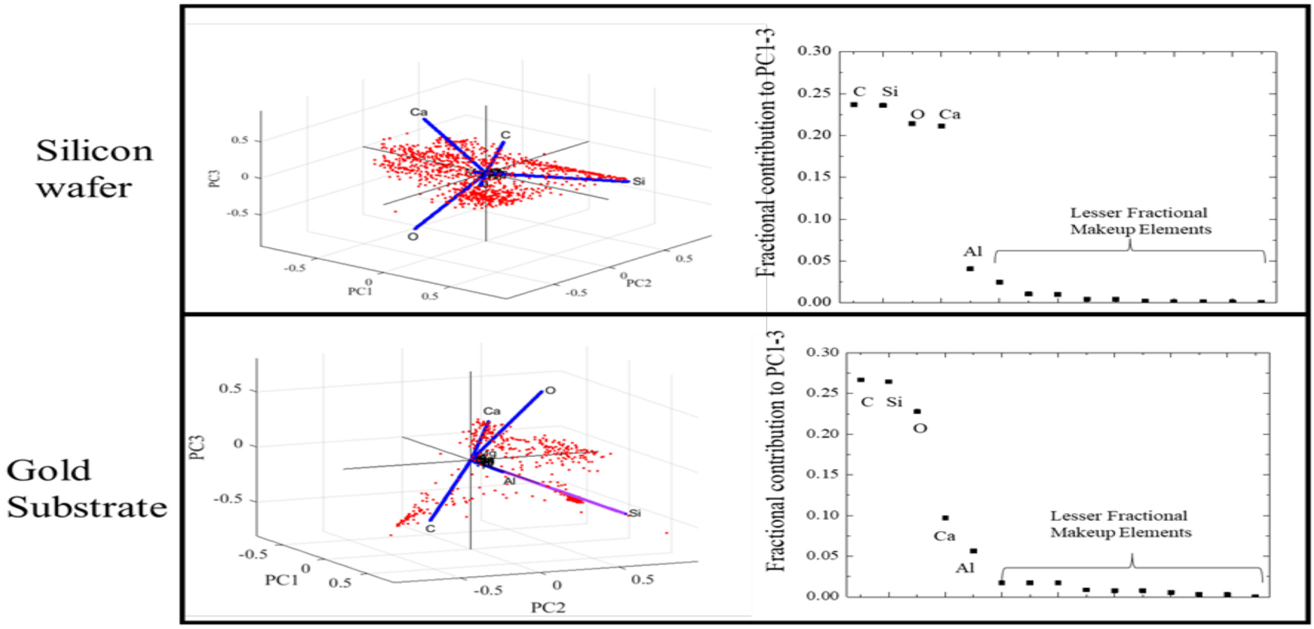

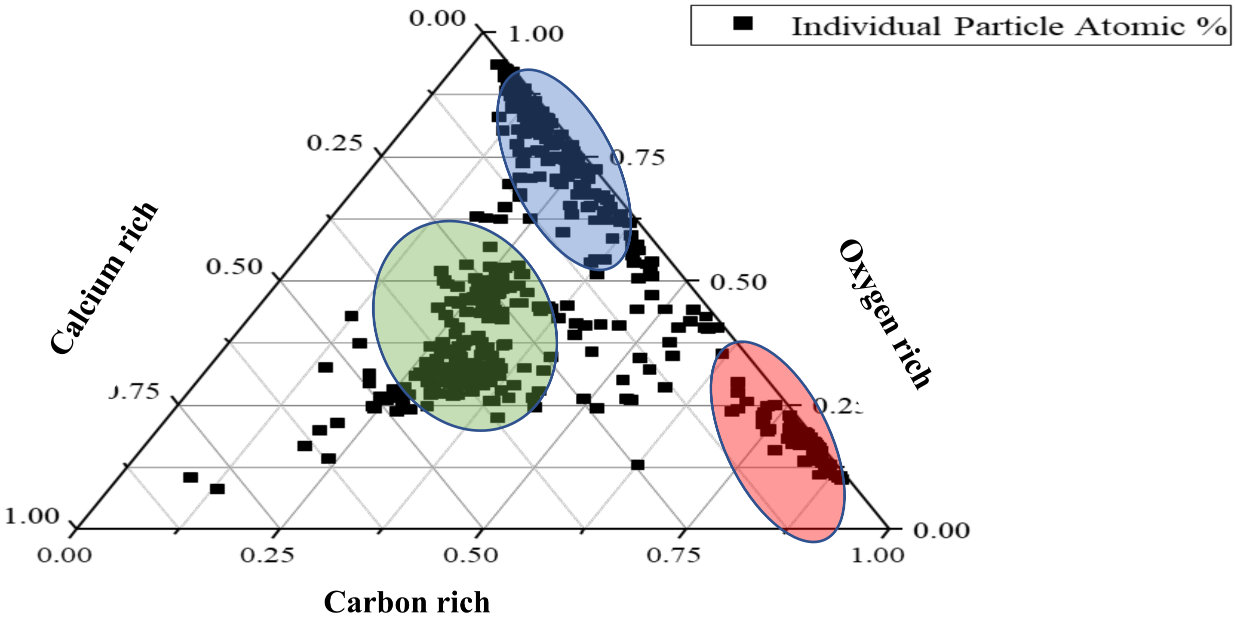

Figure S7: plots showing each element's score (essentially a contribution to the variation) for the first three principal components (PCs 1-3 contribute to >90% of the variation) the plot of the absolute value of the score for the first three principal components is shown on the right. The plot on the bottom is a ternary elemental composition plot showing the grouping of the particles in reduced form.

An alternative analysis was conducted using MATLAB k-clustering analysis. Normalized elemental % data was first analyzed using the silhouette method to determine the number of clusters. The result was consistent with PCA (three cluster types). Centroid locations were calculated and every datapoint assigned to the nearest centroid. Similar results were obtained for both the reduced form of the data (ternary % of C, Ca, O) and the entire elements listed above.

Figure S8: SEM images showing particulate categorized as one of the three types.

Multi- Component Dust (Calcium rich)

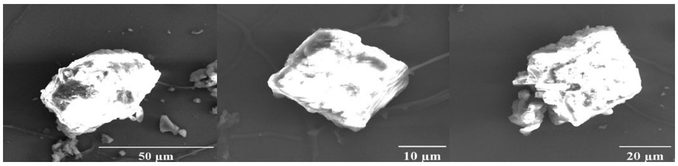

Single Component Dust (oxygen rich)

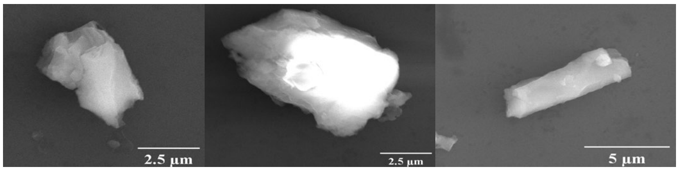

Biotic Activity and Pollen Aggregates
(carbon rich)

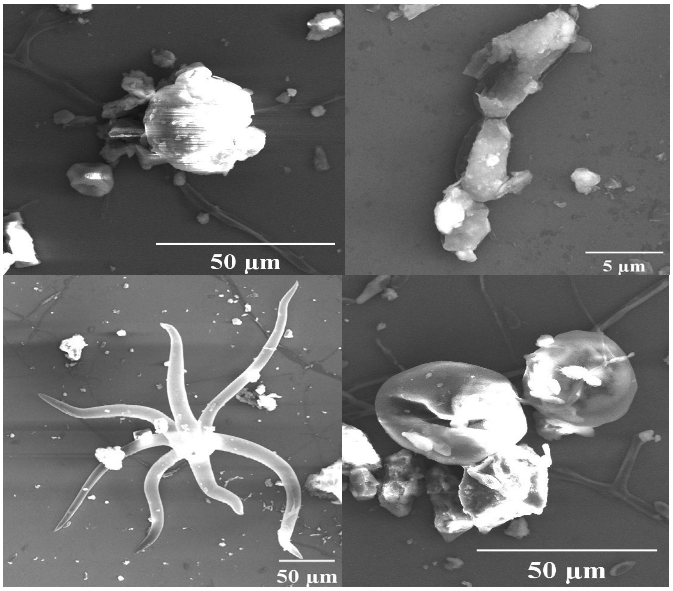

Figure S8: SEM images showing particles classified in each of the three particle types. The top shows multicomponent dust (calcium rich), the middle shows single component (oxygen rich), and the bottom shows the biotic activity (carbon rich). A note is that the carbon-rich particles contain whole and fragmented pollen as well as some fungal growths and leaf stellate.

Figure S9: Histogram showing the variability of four replicates of the CCSEM data.

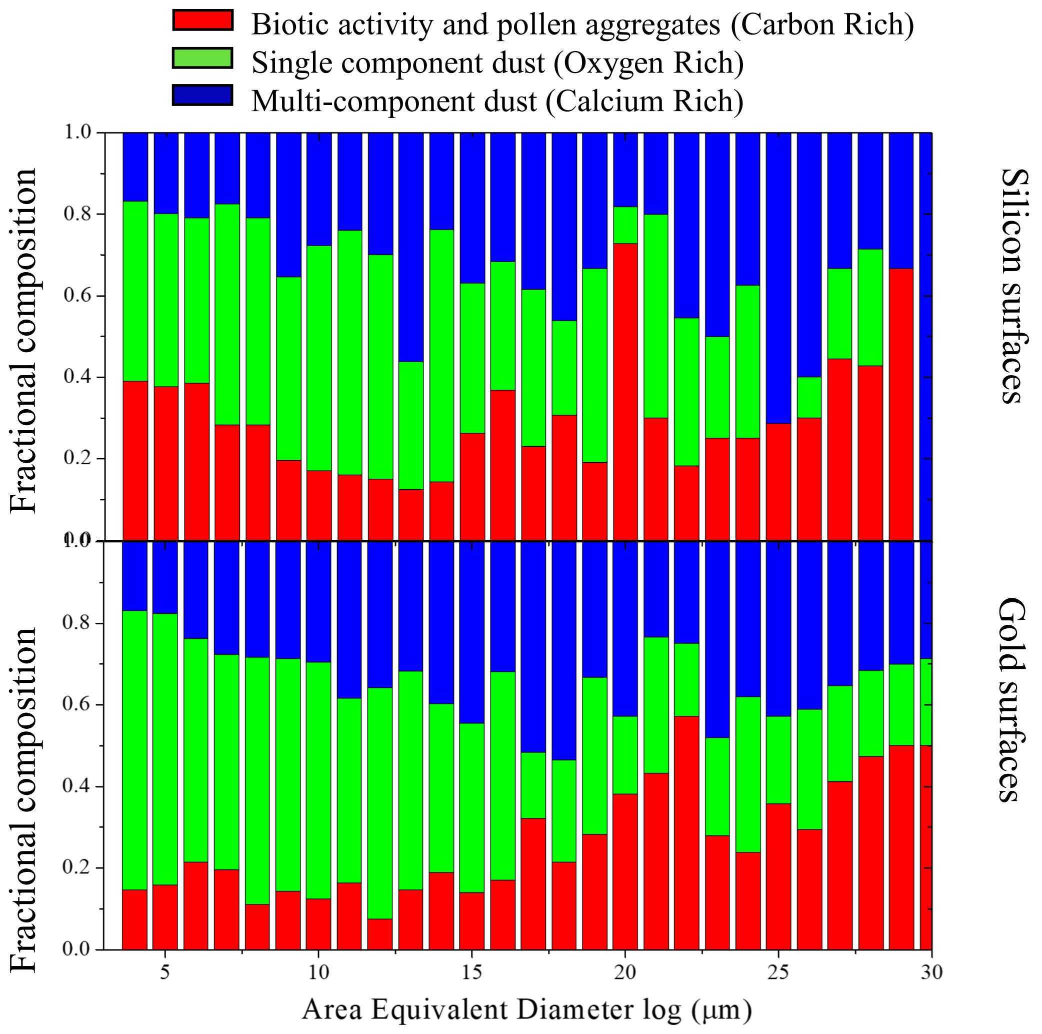

Figure S9: A representation of particle type for films formed on the silicon (top) and gold (bottom) surfaces. The contribution to total particles of each particle type (carbon rich, oxygen rich, or calcium rich) is shown with red, green, and blue bars (respectively).

Figure S10: SEM and EDS mapping of leaf stellate and aggregates

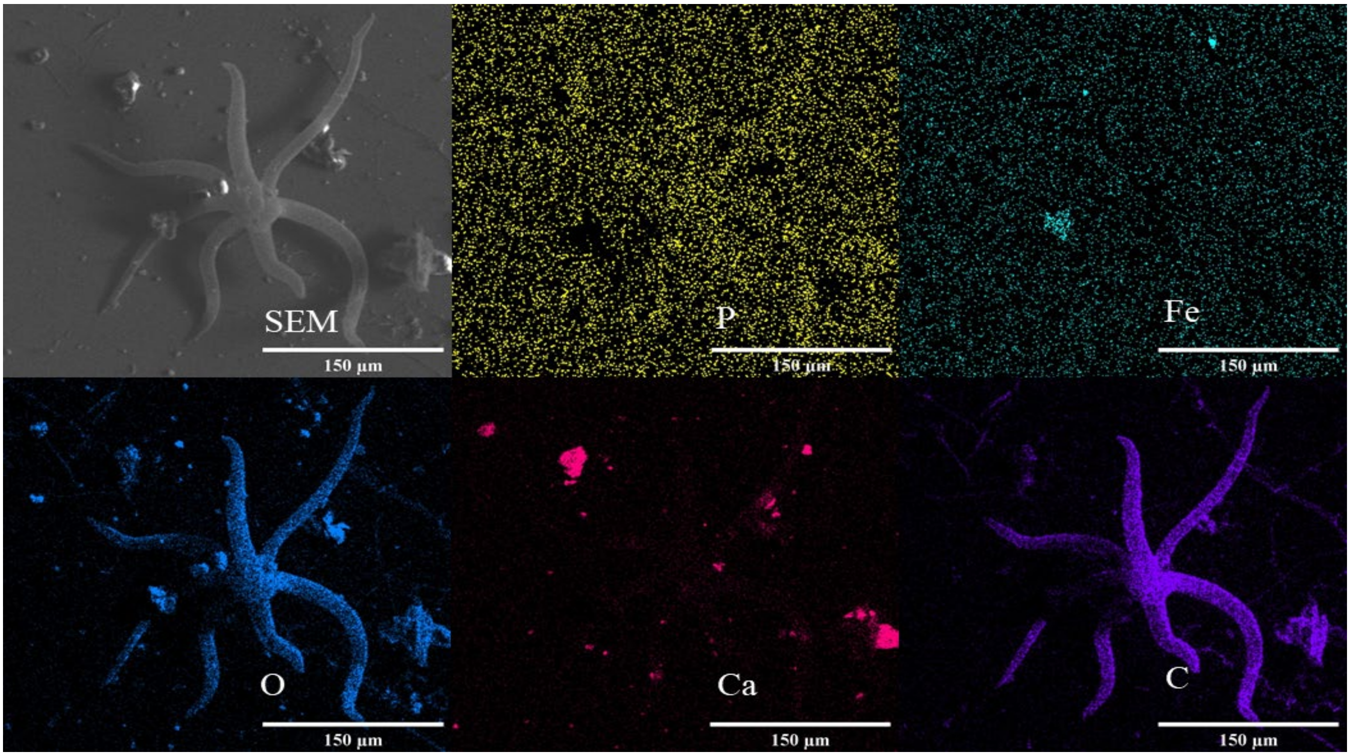

Figure S10: SEM image and elemental mapping showing a leaf stellate we believe is from an oak plant and the aggregated particulate around the central morphology.

Figure S11: SEM and EDS mapping of inorganic particles on silicon wafers

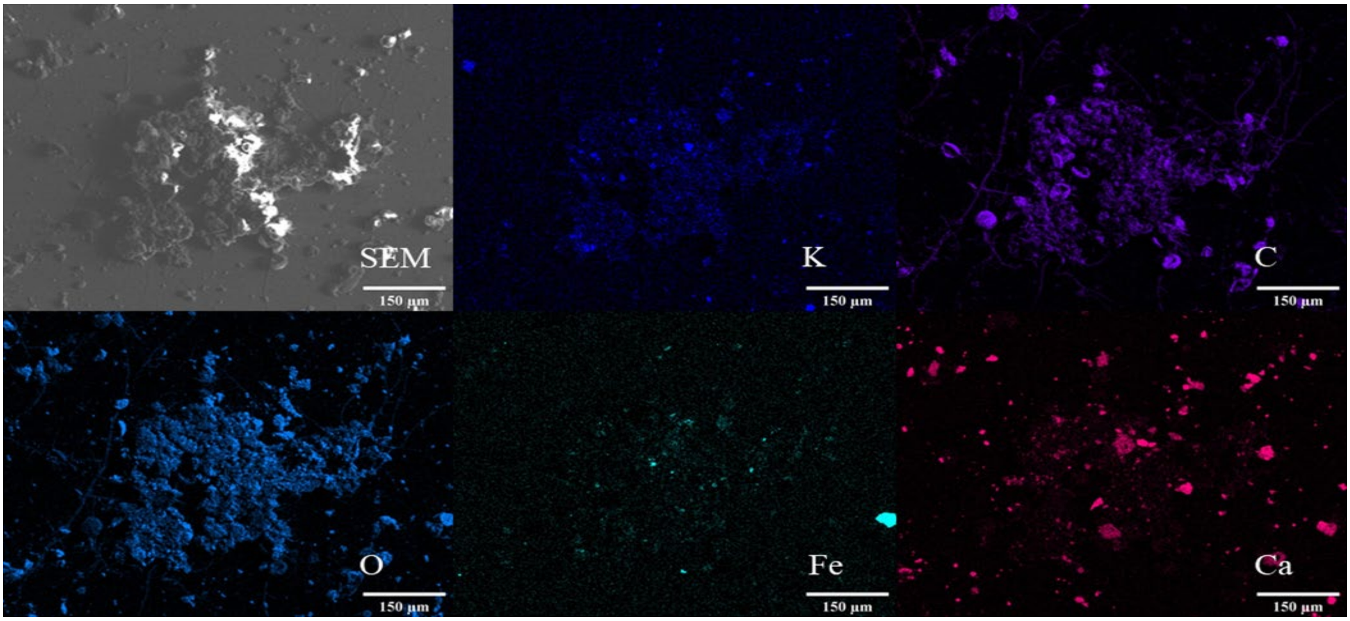

Figure S11: SEM-EDS elemental mapping showing prevalence of calcium oxalate or carbonate, iron, and potassium grouped in a large aggregate on the surface of silicon wafer.

Figure S12: SEM-EDS mapping of the biotic moieties observed

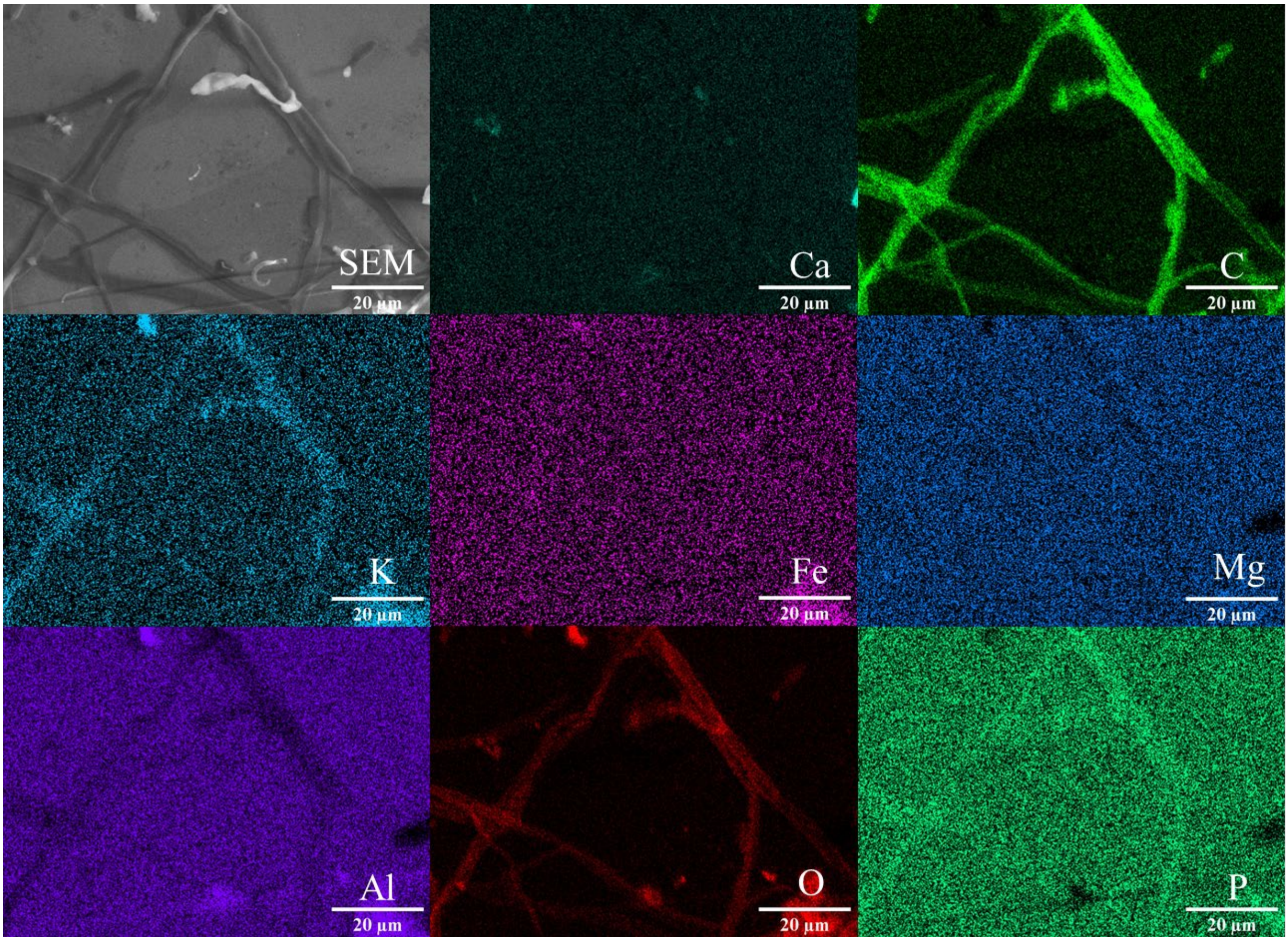

Figure S12: SEM-EDS elemental mapping of the biotic moieties observed.

Figure S13: ToF-SIMS mapping of ions of interest on the gold surface.

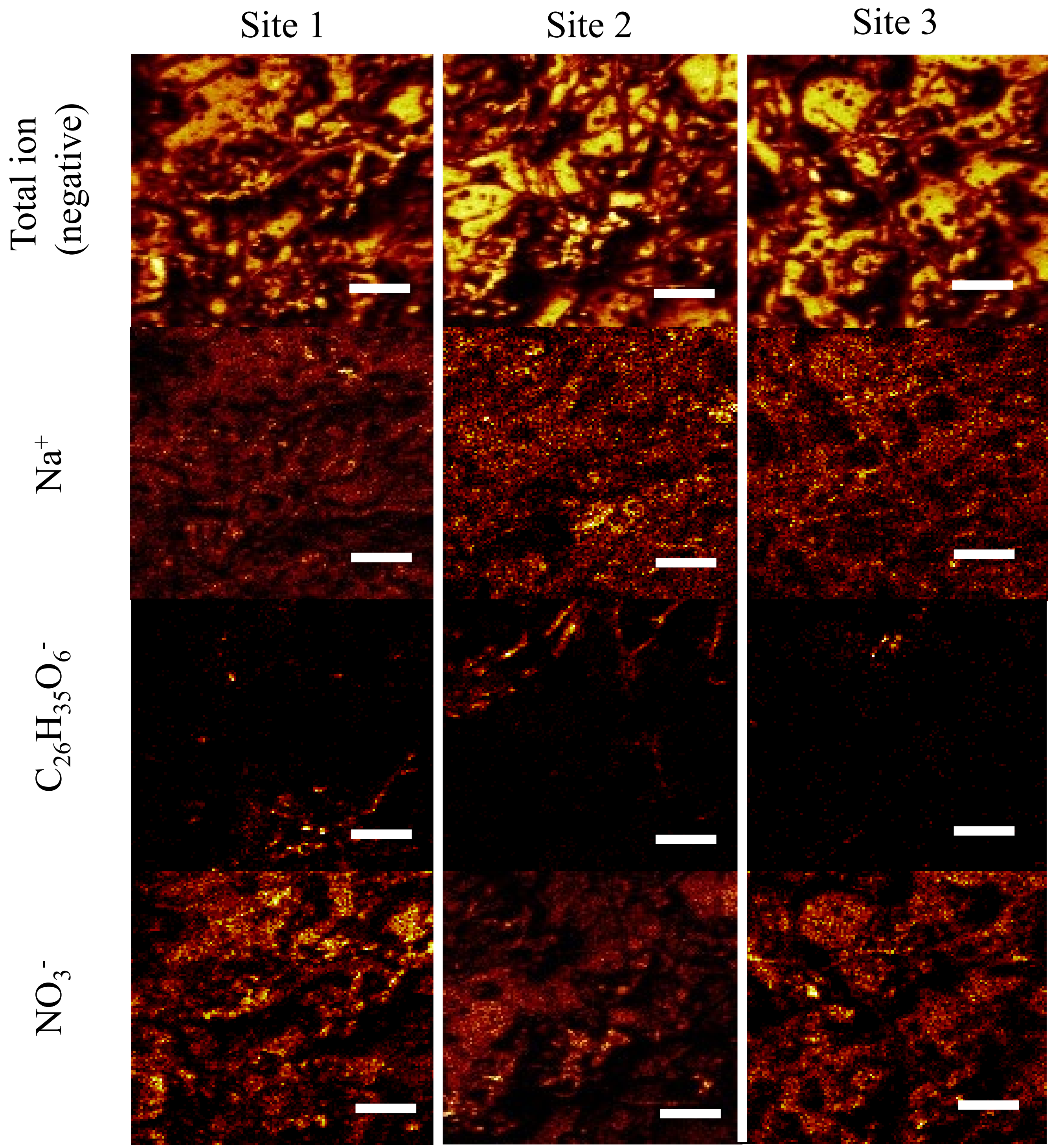

Figure S13: ToF-SIMS imaging showing the heterogeneous distributions of sodium and nitrate. We have also included images of a distinct molecular fragment tracing with the growths of the fungi on the surface. The scalebar is 100μm.

Figure S14: Van Krevelen plot showing the identified species using Nano-DESI.

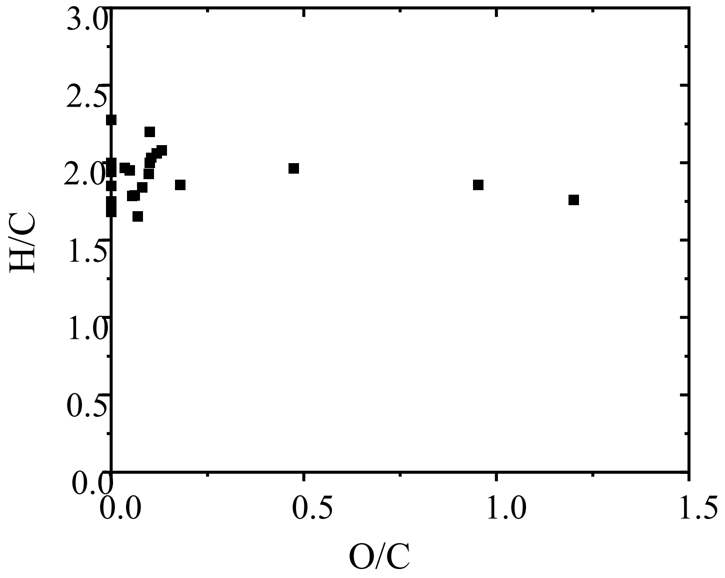

Figure S14: A Van Krevelen plot showing the distribution of the identified ions analyzed using the Nano-DESI technique.
